# Age‐associated changes in long‐chain fatty acid profile during healthy aging promote pro‐inflammatory monocyte polarization via PPARγ

**DOI:** 10.1111/acel.12416

**Published:** 2015-11-02

**Authors:** Chathyan Pararasa, John Ikwuobe, Shahjahan Shigdar, Alexis Boukouvalas, Ian T. Nabney, James E. Brown, Andrew Devitt, Clifford J. Bailey, Stuart J. Bennett, Helen R. Griffiths

**Affiliations:** ^1^ Life & Health SciencesAston UniversityBirminghamB4 7ETUK; ^2^ Aston Research Centre for Healthy AgeingAston UniversityBirminghamB4 7ETUK; ^3^ Engineering & Applied SciencesAston UniversityBirminghamB4 7ETUK; ^4^ The London HospitalLondonUK

**Keywords:** anti‐inflammatory, macrophage, mitochondrial ROS, oleate, oxidative phosphorylation, palmitate

## Abstract

Differences in lipid metabolism associate with age‐related disease development and lifespan. Inflammation is a common link between metabolic dysregulation and aging. Saturated fatty acids (FAs) initiate pro‐inflammatory signalling from many cells including monocytes; however, no existing studies have quantified age‐associated changes in individual FAs in relation to inflammatory phenotype. Therefore, we have determined the plasma concentrations of distinct FAs by gas chromatography in 26 healthy younger individuals (age < 30 years) and 21 healthy FA individuals (age > 50 years). Linear mixed models were used to explore the association between circulating FAs, age and cytokines. We showed that plasma saturated, poly‐ and mono‐unsaturated FAs increase with age. Circulating TNF‐α and IL‐6 concentrations increased with age, whereas IL‐10 and TGF‐β1 concentrations decreased. Oxidation of MitoSOX Red was higher in leucocytes from FA adults, and plasma oxidized glutathione concentrations were higher. There was significant colinearity between plasma saturated FAs, indicative of their metabolic relationships. Higher levels of the saturated FAs C18:0 and C24:0 were associated with lower TGF‐β1 concentrations, and higher C16:0 were associated with higher TNF‐α concentrations. We further examined effects of the aging FA profile on monocyte polarization and metabolism in THP1 monocytes. Monocytes preincubated with C16:0 increased secretion of pro‐inflammatory cytokines in response to phorbol myristate acetate‐induced differentiation through ceramide‐dependent inhibition of PPARγ activity. Conversely, C18:1 primed a pro‐resolving macrophage which was PPARγ dependent and ceramide dependent and which required oxidative phosphorylation. These data suggest that a midlife adult FA profile impairs the switch from proinflammatory to lower energy, requiring anti‐inflammatory macrophages through metabolic reprogramming.

## Introduction

During aging, there is a redistribution of body fat away from subcutaneous stores to other depots including muscle and viscera (Pararasa *et al*., 2014). Visceral adipose tissue is less efficient in storing fatty acid (FA) and there is a concomitant increase in circulating free fatty acids (FFA) that promote insulin resistance leading to elevated blood glucose concentrations and risk for type 2 diabetes (T2DM) (Carlsson *et al*., 2000; Pilz *et al*., 2006). Raised fasting plasma FFA levels are associated with an increase in risk factors for all‐cause and cardiovascular mortality such as blood pressure, impaired endothelial cell function and increased inflammatory markers (Tripathy *et al*., 2003). However, not all FFA exert the same effects. The anti‐inflammatory effects of omega 3 FA have long been reported and, more recently, saturated FAs have been linked causally to inflammation and insulin resistance, acting on pancreatic islets and monocytes in a Toll‐like receptor (TLR) 4‐dependent manner (Huang *et al*., 2012). TLR4‐activated monocytes migrate to the target tissues, directed by a chemotactic gradient of chemokines and stromal factors (Weber *et al*., 2004).

In tissues, monocytes differentiate into two major effector macrophage phenotypes, according to environmental cues, which are classified broadly into (‘M1’) classically activated, inflammatory and (‘M2’) alternatively activated, pro‐resolving phenotypes. Inflammatory monocyte/macrophages phagocytose pathogens and cellular debris, and pro‐resolving monocyte/macrophages secrete wound healing and immunosuppressive factors (Gordon, 2003; Erwig & Henson, 2007; Mosser & Edwards, 2008).

Classically activated M1 macrophages are polarized in response to Th1 cytokines, for example IFNγ or pathogen‐associated molecular patterns (PAMPs), for example LPS (Schwartz & Svistelnik, 2012), and are involved primarily in defence against bacterial or viral pathogens. The M1 phenotype is characterized by elevated production of pro‐inflammatory cytokines TNF‐α, IL‐1β, IL‐6, IL‐8 and IL‐12, accompanied by elevated expression of pattern‐recognition receptors, for example TLR and NOD‐like receptors (Martinez *et al*., 2009). In contrast, alternatively activated M2 macrophages are polarized in response to Th2 cytokines IL‐4/IL‐13, displaying a phenotype which limits inflammation through the production of anti‐inflammatory cytokines such as TGF‐β1, increased surface expression of scavenger receptors, extracellular matrix formation, clearance of apoptotic cells and debris, and in the resolution of inflammation (Devitt & Marshall, 2011; Lopez‐Castejon *et al*., 2011; Takeuch & Akira, 2011).

In metabolic disorders that are increasingly prevalent during aging, there is an increase in the M1/M2 macrophage ratio (Zeyda *et al*., 2007; Chinetti‐Gbaguidi & Staels, 2011). This may be due to the accumulation of visceral adipose tissue during aging; visceral adipose tissue‐derived cytokines recruit monocytes and promote their differentiation into M1 pro‐inflammatory macrophages that are suggested to promote insulin resistance (Lumeng & Saltiel, 2011). Similarly, prediabetic individuals have elevated circulating M1 monocyte phenotypes (Fadini *et al*., 2013). In atherosclerotic plaques, macrophage phenotype associates with plaque stability; M1 are localized in the rupture prone and M2 in the cell‐rich, stable regions, respectively (Stoger *et al*., 2012). Modulation of monocyte/macrophage polarization offers a tangible opportunity to reduce cardiovascular dysfunction in aging, T2DM and obesity (Ghattas *et al*., 2013).

During monocyte differentiation, there is a metabolic switch towards glycolysis in the early initiation of inflammation in M1 cells and oxidative phosphorylation in M2 cells during the late adapting, resolving phases (M2; Liu *et al*., 2012). The principal substrates for glycolysis and oxidative phosphorylation are sugars and FA, respectively. This suggests that an increase in circulating FA substrates may promote inflammatory resolution. However, in T2DM mouse models, pancreatic β‐cells respond to a SFA diet by producing chemokines that recruit CD11b(+)Ly‐6C(+) M1‐type proinflammatory monocytes/macrophages to islets (Eguchi *et al*., 2012) suggesting that FA substrates have differing effects on innate immunity. There are very little data examining the effects of aging on FFA in healthy midlife adults, and to date, the FFA relationship with the monocyte polarization process has not been investigated. It is not known in aging whether the elevated FFA present can affect atherosclerosis risk by influencing monocyte–macrophage phenotype.

Recently, our group demonstrated that a pro‐atherogenic monocyte phenotype could be induced by palmitate (C16:0) via the *de novo* synthesis of ceramides. In contrast, monounsaturated FA (MUFA) prevented this phenotype (Gao *et al*., 2012). Therefore, we have investigated the FA profile associated with healthy aging and examined its relationship to monocyte phenotype, metabolism and polarization.

## Results

### FA profile in healthy aging

Anthropometric and biochemical characteristics of the young [24 (3.76) years; mean standard deviation (SD)] and midlife [58 (6.1) years; mean (SD)] male subjects recruited into this study are described in Table 1. We limited our study to males to exclude the confounding effects of hormonal changes in peri‐/menopausal older women; for example the decline in oestrogen concentration with age associates with monocyte activation, an increase in very low density lipoprotein (VLDL) and triglycerides and decrease of high density lipoprotein (HDL) in menopausal women. All males were medication free and exhibited normal blood glucose, cholesterol, triglycerides, systolic blood pressure and waist‐to‐hip ratio. The body mass index (BMI), waist circumference, diastolic blood pressure, plasma insulin and HOMA‐IR were significantly higher in midlife subjects (*P* < 0.05).

**Table 1:** Baseline characteristics of healthy, fasting male subjects

	Young, *n* = 26		Midlife, *n* = 21		Significance
	Mean	Standard deviation	Mean	Standard deviation	*P*
Age (years)	24.16	3.76	57.53	6.07	<0.0001
Weight (Kg)	76.11	11.97	80.58	10.05	>0.05
Height (m)	1.785	0.71	1.808	0.08	>0.05
BMI Kg m^−2^	22.71	2.88	24.53	2.22	0.038
Waist (cm)	88.92	6.82	98.54	7.07	0.0083
Hip (cm)	99.16	6.93	104.3	5.24	>0.05
Waist:hip ratio	0.8969	0.059	0.946	0.07	>0.05
Systolic blood pressure	123.3	13.65	131.7	13.24	>0.05
Diastolic blood pressure	73.86	10.09	87.06	9.75	0.0006
Heart rate	69.33	13.09	63.92	13.77	>0.05
Glucose (mm)	5.213	0.4	5.447	0.57	>0.05
Total cholesterol (mm)	4.325	1.16	4.8	0.77	>0.05
HDL cholesterol (mm)	1.01	0.22	0.9907	0.3	>0.05
LDL cholesterol (mm)	2.85	1.04	3.453	1.04	>0.05
TG (md dL^−1^)	62.68	0.29	35.95	0.15	>0.05
Insulin (U mL^−1^)	8.588	1.56	12.47	7.18	0.0013
HOMA‐IR	2.015	0.37	2.96	1.42	0.0032

Concentrations of plasma total SFA, MUFA and PUFA in healthy fasting mid‐life male subjects were significantly greater than in younger, healthy male adults (*P* < 0.05). Individual FA concentrations were noted as significantly elevated in midlife adults for SFA C14:0 and C24:0 (*P* < 0.001), the MUFA C14:1, C18:1 and C24:1 (*P* < 0.05) and the PUFA C18:3n6, C22:3n6 and C22:2 (*P* < 0.05; Table 2).

**Table 2:** Plasma fatty acid concentrations in healthy, fasting male subjects

Fatty acids (μm)	Young (*n* = 26)		Midlife (*n* = 21)		*P*
	Median	Interquartile range	Median	Interquartile range	
C14:0	5.86	1.64–18.6	38.7	17.3–78.7	0.0014
C14:1	<LOD	<LOD	12.9	0–52.4	<0.0001
C16:0	186	107.9–243	214	82.8–412	0.74
C16:1	6.99	1.78–15.9	19.8	0–45.5	0.3
C18:0	50.7	31.9–76.7	74.1	46.5–118	0.07
C18:1	130	63.3–327	235	133–470	0.035
C18:2	31.6	6.99–66.2	18.3	11.3–67.3	0.67
C18:3n6	35.6	23.5–73.6	56.6	48.7–120	0.049
C18:3n3	26.1	12.4–61.5	31.4	6.75–77.2	1
C22:2	<LOD	< LOD	<LOD	<LOD–19	0.017
C24:0	7.67	2.87–17.5	33.2	12.55–83.4	0.0024
C24:1	12.3	6.26–43.0	52.7	21.7–112	0.0097
C22:6n3	<LOD	< LOD–4.02	5.4	<LOD–17.3	0.034

### Aging oxidative and inflammatory phenotype

**Figure 1:**
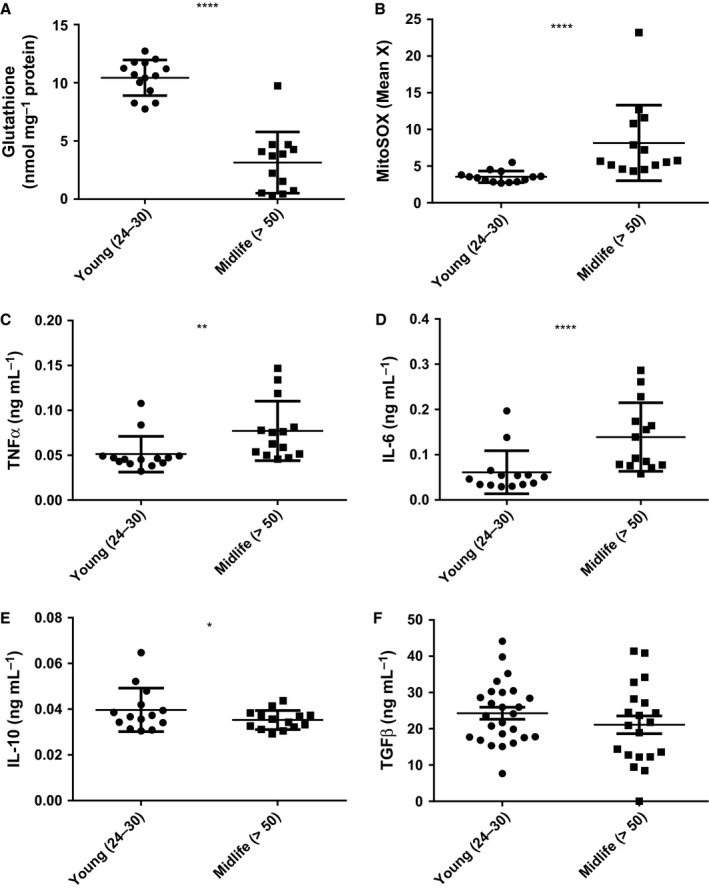
Oxidative stress and inflammation increase with age. Blood was from healthy, consenting male donors (years), (A) Total plasma glutathione was determined by the recycling assay and corrected for plasma protein (mg) determined by the bicinchoninic acid assay (Gao *et al*., 2012). (B) Oxidation of MitoSOX Red in mononuclear cells was by flow cytometry in whole‐blood samples after erythrocyte lysis using Optilyse C. (C–F) Plasma cytokine analysis (TNF‐α, IL‐6, TGF‐β1 and IL‐10) was by ELISA. Data are presented as means and standard deviation. Mann–Whitney *U*‐tests showed *P < 0.05, **P < 0.01 and ****P < 0.0001.

Samples were processed for oxidative stress biomarkers within 5 min of blood collection. Mononuclear cell oxidation of MitoSOX Red was elevated significantly, and plasma total glutathione concentrations were decreased significantly in midlife subjects (*P* < 0.001; Fig. 1A,B).

Plasma pro‐inflammatory cytokines (TNF‐α and IL‐6) were elevated significantly in healthy midlife subjects (*P* < 0.01), whereas the concentration of the anti‐inflammatory cytokine IL‐10 was lower (*P* < 0.05) and TGF‐β1 was not different between groups (Fig. 1C–F). These findings are consistent with pro‐oxidative and pro‐inflammatory effects of aging.

### FAs as predictors of age and plasma cytokines

Multiple‐regression models were generated to predict age and plasma cytokine concentrations. Very long‐chain FA C24:0, C24:1 and C18:3n6 with diastolic blood pressure, cytokines and lipids predicted age (*P* < 0.001; Table 3). The major FA predictor of plasma TNF‐α was C16:0 (*P* < 0.001; Table S1, Supporting Information). Of the parameters tested, plasma IL‐6 concentration was predicted by FA only, including the PUFA, C18:3n6 and C18:3n3 and the SFA C14:0 and C24:0 (*P* < 0.001; Table S2). High plasma TGF‐β1 concentration was predicted by low plasma C24:0 and C18:0, low low‐density lipoprotein (LDL) cholesterol, age and high C18:3n6 (*P* = 0.014; Table S3). None of these analytes predicted plasma IL‐10 concentration.

**Table 3:** Multiple linear regression model predicting age

Variable	Standardized coefficient (β)	*P*
Intercept		0.251
C24:0	0.829	<**0.001**
Diastolic blood pressure	0.508	**0.003**
C24:1	0.45	**0.003**
TNF‐α	0.371	**0.034**
Triglyceride	0.344	**0.011**
TGF‐β1	0.259	**0.054**
HDL cholesterol	−0.294	**0.023**
C18:3n3	−0.382	**0.009**
IL‐6	−0.575	**0.01**

Fatty acid that correlated significantly with age in a univariate regression model were fitted into a multiple‐regression model, adjusting for waist circumference and cytokines. Model summary: *R* = 0.934, *R* ^2^ = 0.873, adjusted *R* ^2^ = 0.778, *P* < 0.001. Bold values represent *p* values determined through multiple regression analysis.

### FA prime monocytes for cytokine secretion according to saturation not chain length

**Figure 2:**
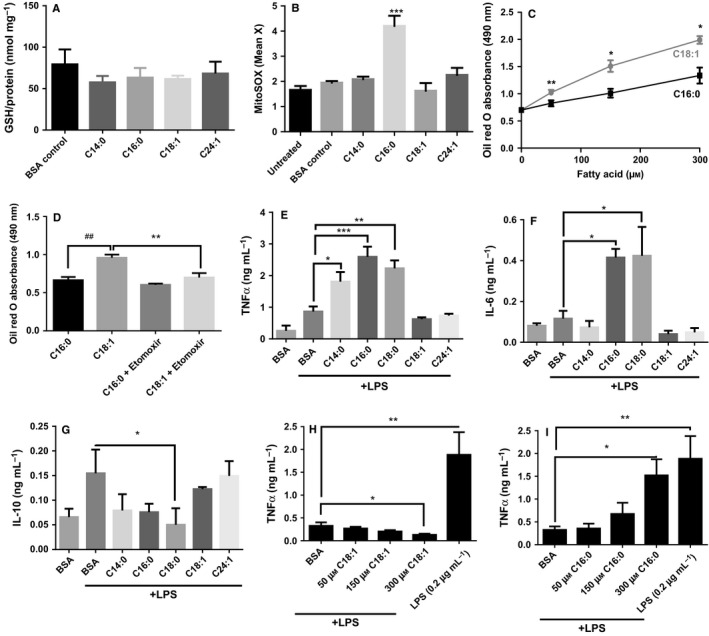
Fatty acids elicit oxidative stress and prime monocytes for cytokine secretion according to saturation not chain length. THP‐1 monocytes were incubated for 24 h with 150 μm fatty acid (FA) conjugated to FA‐free bovine serum albumin (BSA). (A) Cellular glutathione was determined by the recycling assay and corrected for protein determined by bicinchoninic acid assay; (B) oxidation of MitoSOX Red was measured by flow cytometry. (C) The concentration‐ and FA‐dependent accumulation of lipids in monocytes over 24 h; and (D) the effect of etomoxir on lipid storage in monocytes after 50 μm FA was measured by Oil Red O staining and quantitated spectrophotometrically at 490 nm. THP‐1 monocytes pretreated with BSA ± FA for 6 h prior to activation by 5 ng mL^−1^ LPS for 18 h; effect on TNF‐α (E), IL‐6 (F) and IL‐10 (G) was determined by ELISA. Concentration‐dependent effect of C16:0 (H) and C18:1 (I) on TNF‐α secretion after LPS activation (5 ng mL^−1^). 0.2 μg mL^−1^ LPS was used as a positive control to induce maximal cytokine secretion. Data are presented as mean + SEM from three independent experiments. Data were analysed by ANOVA or paired *t*‐test analysis as appropriate compared to BSA control; *P < 0.05, **P < 0.01 and ***P < 0.001 and ## represents *P* < 0.01 compared with C16:0.

We have shown previously that the C16:0 and C18:1, the major SFA and MUFA in plasma induce a pro‐ and anti‐inflammatory phenotype, respectively, as indicated by cell surface antigen expression in monocytes (Gao *et al*., 2012). Here, we investigated whether the major FAs C16:0 and C18:1 or the FAs elevated ≥ fivefold with age (C14:0 and C24:1) influenced redox state and monocyte cytokine production *in vitro*. FAs are transported *in vivo* on albumin and for the purposes of our experiments were delivered by bovine serum albumin (BSA); therefore, BSA was used as vehicle control throughout. The presence of BSA had no effect compared to untreated control (Fig. 2A). None of the cell treatments had any significant effect on cell viability (data not shown).

While there was no difference in effect between SFA and MUFA on cellular glutathione concentration, only C16:0 significantly increased oxidation of MitoSOX Red over 24 h (*P* < 0.001; Fig. 2A,B). Addition of MUFA increased triglyceride storage as shown by Oil Red O staining (*P* < 0.05; Fig. 2C) in a manner that was inhibited by the diacylglycerol transferase inhibitor etomoxir (Fig. 2D). However, in response to increasing FA concentrations, cytokine secretion by THP1 monocytes was not altered (Fig. S1, Supporting Information). Therefore, we examined whether the presence of FAs may affect monocyte response to an activating stimulus of LPS. The following experiments were conducted to investigate the effects of FA in the presence of a submaximal LPS concentration.

Prior to activation with LPS (5 ng mL^−1^) which induces submaximal cytokine responses, THP1 monocytes were pretreated for 6 h with BSA, SFA or MUFA. SFA C16:0 and C18:0 increased IL‐6; C14:0, C16:0 and C18:0 increased TNF‐α secretion; and C18:0 inhibited IL‐10 secretion (*P* < 0.01). In contrast, C18:1 did not exert any priming effect on LPS‐induced TNF‐α or IL‐10 secretion, nor did it inhibit the secretion of IL‐10 by monocytes after LPS activation (Fig. 2E–G). To investigate the contribution of reactive oxygen species (ROS) and NFkB activation to the SFA priming effect, monocytes were incubated with MnTBAP and SN50, respectively. Scavenging ROS and inhibiting NFκB prevented the priming effects of SFA (Fig. S2). Closer examination of the major plasma MUFA C18:1 and SFA C16:0 confirmed that their effects on LPS‐induced TNF‐α secretion were dose dependent (Fig. 2H,I) with C16:0 increasing the sensitivity to LPS to near‐maximal levels of cell activation. These findings support the hypothesis that saturation is a major driver for the inflammatory effects of FA.

### Specific FAs recapitulate polarization effects of cytokines during monocyte polarization to macrophages in a ceramide‐dependent manner

**Figure 3:**
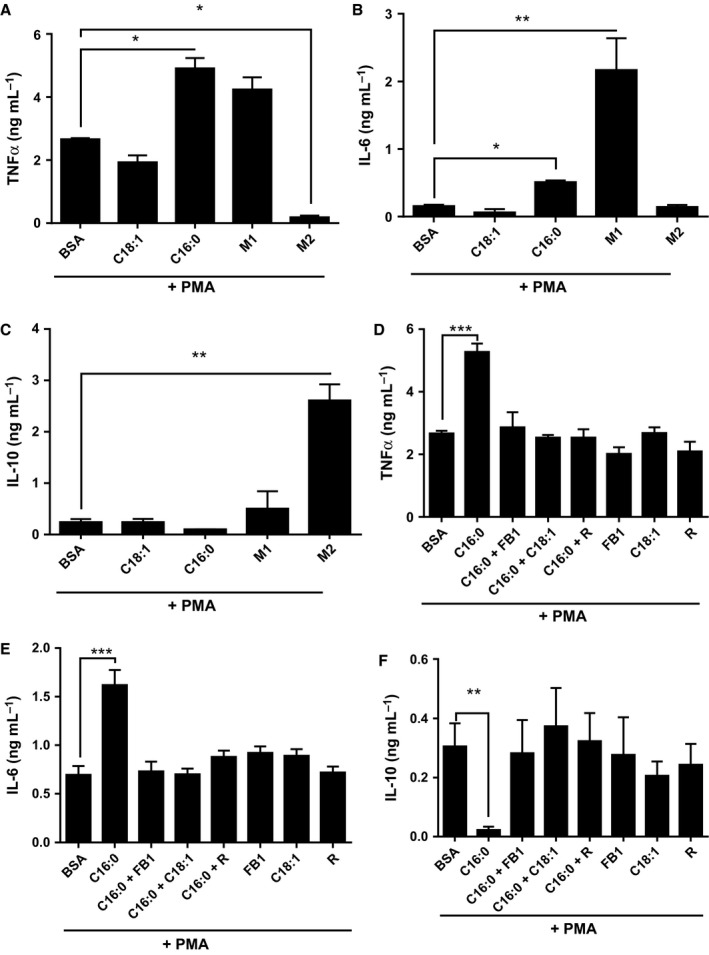
Pretreatment with palmitate C16:0 induces a pro‐inflammatory macrophage expression profile in phorbol myristate acetate (PMA)‐differentiated THP‐1 cells. THP‐1 monocytes were primed (6 h) with C16:0 or C18:1 (300 μm), M1 macrophage‐cytokine cocktail (IFNγ+LPS) or M2 macrophage‐cytokine cocktail (IL4 + IL13) prior to differentiation with 320 nm PMA (18 h). Secreted TNF‐α (A, D), IL‐6(B, E), IL‐10 (C, F) were determined by ELISA in the presence and absence of fumonisin B1 (FB1) and rosiglitazone (R). Data is mean and SEM from three independent experiments analysed by ANOVA; ***P < 0.001, **P < 0.01, and *P < 0.05 vs. appropriate bovine serum albumin control.

TNF‐α and IL‐6 secretion were up to tenfold higher from M1 than M2 THP‐1 macrophages, but IL‐10 secretion by M1 was only 15% of that from M2 cells (Fig. 3A–C; *P* < 0.05). The M1‐like phenotype was mimicked by C16:0 priming. In contrast, C18:1 showed a trend to inhibit TNF‐α and IL‐6 secretion compared to phorbol myristate acetate (PMA) alone but did not affect PMA‐induced IL‐10 secretion. To better understand the mechanism associated with C16:0‐induced M1 polarization, we determined whether lipid metabolism might be a contributory factor. In the presence of fumonisin B1 (FB1) to prevent ceramide formation, rosiglitazone as a PPARγ agonist and C18:1 as a competitor for metabolic carrier molecules such as CoA, the C16:0‐mediated priming effect towards an M1 phenotype was abolished (Fig. 3D,E; *P* < 0.001). Conversely, the inhibitory effect of C16:0 on IL‐10 secretion by PMA‐differentiated cells was abolished by FB1, C18:1 and rosiglitazone (Fig. 3F).

### Cellular metabolism mediates macrophage polarization

**Figure 4:**
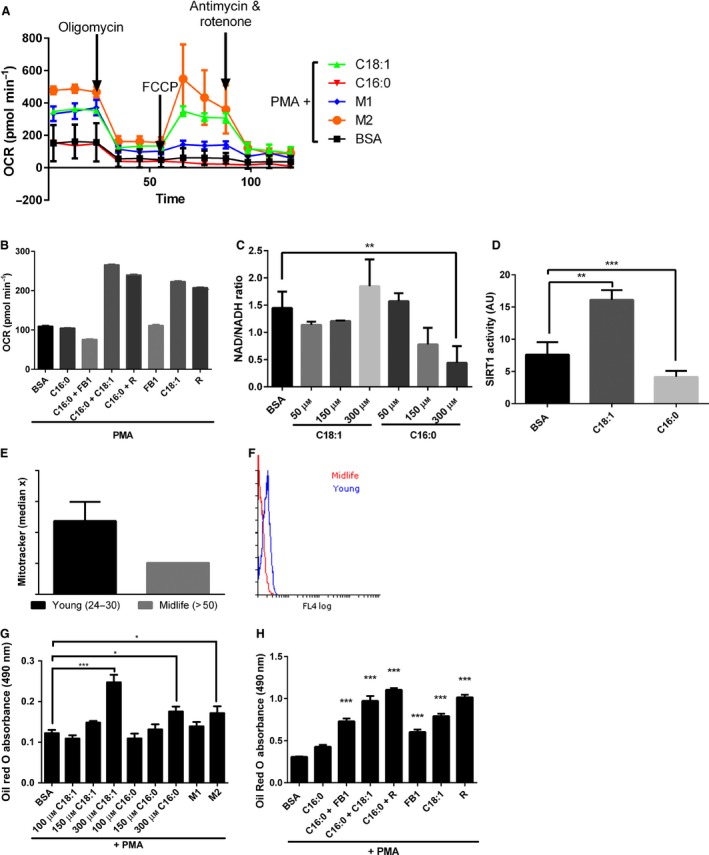
Cellular metabolism mediates macrophage polarization. THP‐1 monocytes were primed (6 h) with C16:0 or C18:1 (50–300 μm), M1 macrophage‐cytokine cocktail (IFNγ + LPS)or M2 macrophage‐cytokine cocktail (IL4 + IL13) prior to differentiation with 320 nm phorbol myristate acetate (PMA) (18 h). (A) Oxygen consumption rate (OCR) was measured in primed, PMA‐differentiated THP1 cells in the MitoStress test and **(**B) basal oxygen consumption rate after FA priming in the presence and absence of rosiglitazone (R) and fumonisin B1 (FB1) using the Seahorse Analyzer. (C) FA effect on cell NAD ^+^/NADH ratio over 24 h was analysed using a kit from Abcam. (D) SIRT1 activity was measured in nuclear extracts after FA priming (24 h) using a kit from Sigma. (E) Mitochondrial density in monocytes from young (*n* = 3; 24–30 years of age) vs. midlife (*n* = 3; 50–55 years of age) subjects was measured by flow cytometry using MitoTracker. (F) Representative flow histogram overlay of young (24–30) and midlife (> 50) MitoTracker fluorescence. (G) FA concentration‐dependent priming effects (6 h) and (H) in the presence and absence of R and FB1 over 24 h on triglyceride content measured by Oil Red O staining. Data are mean and SEM from three independent experiments. One‐way ANOVA showed ***P < 0.001, **P < 0.01 and *P < 0.05 vs. appropriate bovine serum albumin control.

It has been reported previously that during monocyte differentiation, there is a metabolic switch towards glycolysis in the early initiation of inflammation in M1 cells and oxidative phosphorylation in M2 cells during the late adapting, resolving phase in a SIRT1‐ and 6‐dependent manner (Liu *et al*., 2012). We examined whether FA saturation affected metabolic switching. Consistent with previous reports, we observed that M1 macrophages were unaffected by the MitoStress test, whereas M2 macrophages relied more on oxidative phosphorylation [oxygen consumption rate (OCR); Fig. 4A]. After priming with C18:0, activation by PMA induced THP‐1 macrophages that were more glycolytic measured as extracellular acidification (data not shown) than C18:1‐primed THP‐1 macrophages which had a metabolic profile consistent with M2 macrophages, that is dependent on oxidative phosphorylation. The requirement for PPARγ in metabolic switching was confirmed by the increase in OCR for C16:0 primed macrophages in the presence of rosiglitazone (R). In addition, co‐incubation of C16:0 with an equal concentration of C18:1 during priming resulted in greater OCR dependence in macrophage basal OCR (Fig. 4B). Moreover, C16:0 caused a dose‐dependent depletion of NAD^+^, and SIRT1 activity was inhibited (Fig. 4C,D; *P* < 0.01). In contrast, monocyte activation after priming by C18:1 led to an increase in SIRT1 deacetylase activity (Fig. 4D; *P* < 0.001).

Using MitoTracker™ Green FM to measure mitochondrial density independently of membrane potential, we observed that M2 macrophages showed significantly greater MitoTracker™ Green staining than M1 macrophages (data not shown) and that in CD14+ leucocytes from the peripheral blood of healthy adults, monocyte mitochondrial content was lower in midlife adults compared to younger adults (Fig. 4E,F).

As previously identified in monocytes (Fig. 2), incubation with C18:1 but not C16:0 favoured triglyceride storage. The same dose‐dependent effect was observed in macrophages; M2 macrophages and C18:1‐primed macrophages preferentially stored triglycerides. Furthermore, co‐incubation with rosiglitazone, FB1 and C18:1 significantly increased triglyceride storage in C16:0‐treated cells (Fig. 4G,H). These studies support the hypothesis that FAs differentially modulate lipid metabolism.

### FA‐dependent monocyte polarization is mediated by PPARγ

**Figure 5:**
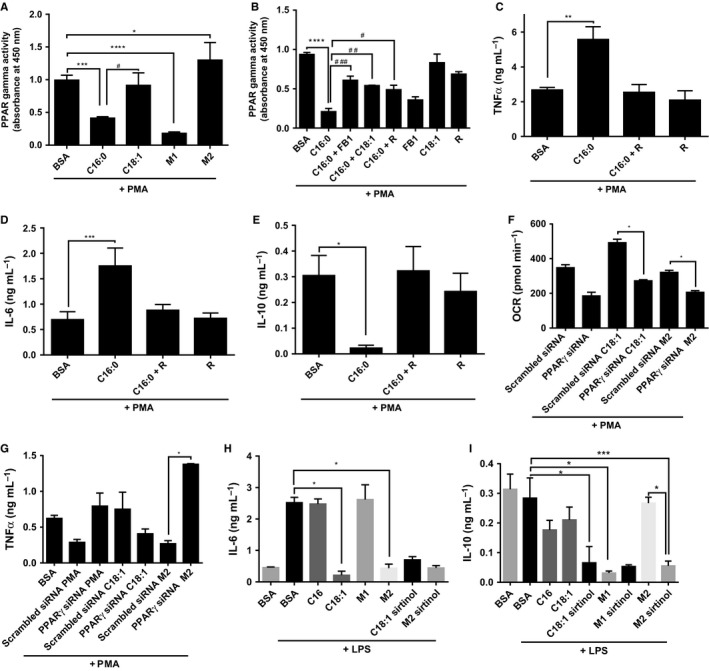
PPARγ controls metabolic switching and inflammatory cytokine secretion and is suppressed by C16:0 and M1 differentiation in macrophages. (A) PPARγ activity was measured in nuclear extracts from THP1 monocyte/macrophages primed by C16:0 and C18:1 (300 μm), and M1 and M2 cytokine cocktails for 6 h before differentiation with 320 nm phorbol myristate acetate (PMA) (18 h) using a TransAM kit. (B) Effect of C16:0 (300 μm; 6 h)‐priming of monocytes ± FB1 (25 μm), rosiglitazone (20 μm) and C18:1 (300 μm), plus 320 nm PMA differentiation (18 h) on TNF‐α (C), IL‐6 (D) and IL‐10 (E) by ELISA. After 24 h transfection with PPARγ small inhibitory (si)RNA or scrambled (scr)siRNA, FAs (6 h) and PMA (18 h) were added, then (F) oxygen consumption rate (OCR) was measured by Seahorse and (G) TNF‐α secretion by ELISA. Effect of SIRT inhibition was measured on (H) IL‐6 and (I) IL‐10 by ELISA. Data are mean + SEM of three independent experiments and analysed by one‐way ANOVA where *P < 0.05, **P < 0.01, ***P < 0.001 and ****P < 0.0005 against bovine serum albumin and #*P* < 0.05, ##*P* < 0.01 and ###*P* < 0.001 against C16:0.

Activity of the upstream lipid metabolism regulator, PPARγ is modulated by acetylation (Qiang *et al*., 2012). We isolated the nuclei of FA‐primed monocyte–macrophages and measured PPARγ activation. C16:0 and M1‐differentiated macrophages showed lower PPARγ consensus sequence binding activity than the PMA‐alone‐differentiated macrophages (Fig. 5A; *P* < 0.001). In contrast, the M2 macrophage‐ binding was significantly increased (*P* < 0.05). Nuclear proteins from M1 macrophages to PPARγ consensus sequence was significantly decreased compared to nuclear proteins from PMA‐only‐primed macrophages (*P* < 0.05).

PPARγ binding activity in C16:0‐treated cells was increased from 15% to more than 50% of PMA‐control activity in the presence of FB1, C18:1 or rosiglitazone (*P* < 0.01; Fig. 5B). PPARγ activation was responsible at least in part for the metabolic switch, as PPARγ siRNA transfected into PMA‐control and C18:1 primed monocyte–macrophages, and inhibited the switch towards oxidative phosphorylation (Fig. 5F).

Acetylated PPARγ is less active in binding CBP (CREB binding protein; co‐localises with PGC1alpha to facilitate PPAR transcription activity) and so promotes NFκB interaction. Therefore, we reasoned that when SIRT1 activity was inhibited by C16:0, acetylation of PPAR**γ** would increase NF‐κB‐dependent cytokine production. Alternatively, NF‐κB‐dependent cytokine production would be increased by PPARγ siRNA. We investigated this experimentally and knocked down PPARγ with siRNA (Fig. S4). TNF‐α and IL‐6 cytokine secretion by C16:0‐primed macrophages was suppressed by rosiglitazone, whereas rosiglitazone promoted IL‐10 secretion (Fig. 5C–E). Furthermore, OCR indicative of oxidative phosphorylation was inhibited, whereas TNF‐α secretion was significantly enhanced from C18:1 primed and M2 macrophages when PPARγ was silenced (Fig. 5F,G). The inhibitory effect of C18:1 on IL‐6 secretion was not significantly affected when SIRT was inhibited; however, IL‐10 secretion from M2 macrophages and C18:1‐primed macrophages was inhibited by sirtinol (Fig. 5H,I). These findings suggest that increased PPARγ acetylation in the presence of FAs contributes to the inflammatory state.

## Discussion

Healthy adults > 50 years of age have increased circulating plasma FFA, and their plasma lipid profile is skewed towards longer FA chains. Here, we have confirmed that inflammatory cytokines are elevated, and anti‐inflammatory cytokine concentrations are lower in the midlife group. In a multivariate regression model, SFAs, particularly long‐chain SFA, predict age and inflammation.

The waist circumference (an index of visceral adipose tissue storage), BMI, diastolic blood pressure, plasma insulin and HOMA‐IR are significantly higher in FA adults than younger controls. An increase in insulin resistance is commonly observed in people aged over 50 years. This leads to elevated circulating concentrations of insulin, glucose and FFA. In our study, all volunteers were fasting and diet diary analysis suggested that fat intake was not different in midlife adults (Fig. S3). Therefore, an elevation of circulating FA is most likely to be a consequence of altered cellular uptake, metabolism and biosynthesis. Others have confirmed that methylation of the FA elongase 2 (*ELOVL2*) gene is altered during aging (Garagnani *et al*., 2012). In independent studies, three of the four most significantly altered CpG methylation sites were within the coding region of *ELOVL2* (Florath *et al*., 2014).

Of the FA that were analysed here, many were significantly increased in midlife (i.e. C14:0, C14:1, C18:1, C18:3n6, C22:2; C22:6n3, C24:0 and C24:1), which are products of ELOVLs 3, 5 and 6 (Guillou *et al*., 2010). Studies of ELOVL6 knockout mice have confirmed a crucial role for ELOVL6 in the development of insulin resistance (Matsuzaka *et al*., 2007), but its activity and relationship to plasma long‐chain FA has not been studied in human aging. One recent report has highlighted the relationship between longevity and long‐chain FA in other species; longer‐lived species had lower concentrations of circulating long‐chain FFA (Jove *et al*., 2013). Here, three of the five FAs that are downstream of ELOVL6 were increased significantly in midlife although FA concentrations did not correlate with HOMA‐IR in healthy adults. An investigation of the relationship of these FA as predictors of insulin resistance in obesity and prediabetes may provide insight into the importance of ELOVL6 activity for insulin resistance in humans. In addition to higher concentrations in MUFA and SFA from older adults, the total PUFA was also significantly higher. The greatest difference was observed for C18:3n6, whereas C18:3n3 concentrations were not different between the two groups; these findings support previous reports of a decline in delta 6 desaturase activity with age. We did not detect free arachidonic acid; an important precursor of pro‐inflammatory prostaglandins. Others have measured arachidonic acid in plasma but included those esterified in cholesterol and triglyceride rather than FFA (Abdelmagid *et al*., 2015). Our subjects were fasting; others have shown that arachidonic acid is retained in triglycerides, phospholipids and cholesteryl esters during fasting (Cunnane, 1990).

We have shown previously that SFA but not MUFA uptake is associated with an increase in monocyte mitochondrial superoxide anion radical concentration in the absence of any stimulus (Gao *et al*., 2012). In the healthy mid‐life adults, oxidation of MitoSOX Red was increased in peripheral whole‐blood mononuclear cells and glutathione was depleted. SFAs may therefore contribute to redox regulated protein activity via mitochondrial ROS. In contrast, MUFAs are less likely to cause redox changes at the mitochondrion and are resistant to peroxidation. The observed low level of FA unsaturation in tissues from long‐lived animals has been suggested to reduce risk of oxidative damage to lipid, DNA and protein (Pamplona *et al*., 2004). This raises a paradox with our data where the older adult population consistently exhibited higher plasma concentrations of both MUFA and SFA. One potential explanation of these findings is that the intracellular pathways which are activated by MUFA may become less sensitive with age, in a similar manner to the observed insulin resistance and decrease in sensitivity to glucose that is observed in aging. Consequently, a skew towards a higher ratio of MUFA:SFA may be required for anti‐inflammatory pathways to be activated in older adults.

Activation of the major inflammatory transcription factor NFκB is redox dependent and likely predisposes to inflammaging (Martinez‐Hervas *et al*., 2010). We identified that SFA were able to prime the pro‐inflammatory monocyte response to LPS, and this effect was knocked down by SN50 and was inhibitable by MnTBAP. This suggested that a ROS‐dependent target upstream of NFκB was important for regulating transcription of inflammatory cytokines in older adults.

Previously, we have shown that C16:0 but not C18:1 increases cytosolic ceramides in monocytes (Gao *et al*., 2012). C16:0 affects the efficiency of the electron transport chain and disrupts the NAD^+^:NADH ratio (Loskovich *et al*., 2005). Consistent with these previous reports, here we observed that in monocytes, NAD^+^ was depleted by C16:0 but not C18:1. The NAD^+^‐dependent SIRT family have been implicated in aging. In adipocytes, the regulation of PPARγ activity by SIRT has been shown to affect cellular lipid metabolism and because there is a close relationship in gene expression profile between adipocytes and monocytes, we reasoned that PPARγ may be an important target in monocytes (Charriere *et al*., 2003). Our studies have shown that PPARγ activity is differentially regulated by FAs with C18:1 acting as a PPARγ agonist. In contrast, C16:0 suppressed basal PPARγ activity. Others have shown that activation of PPARγ by its traditional agonist rosiglitazone reduces cell proliferation rate in U937 monocytes (Wang *et al*., 2011b). Furthermore, rosiglitazone suppressed the expression TLR4 and decreased the production of TNF‐α following LPS treatment (Wang *et al*., 2011a).

Consistent with these findings, the storage of triglycerides was increased significantly in the presence of C18:1. C18:1 is preferentially directed towards lipid storage and mitochondrial metabolism with associated ROS production may be less likely because of increased mitochondrial efficiency. Previous related work has shown in adipocytes that PPARγ senses incoming nonesterified long‐chain FAs and induces storage of long‐chain FAs as triglycerides (Nakamura *et al*., 2014). Others have reported that increasing diacylglycerol acyl transferase 1 (DGAT1) expression increases murine macrophage capacity for TG storage, protects against FA‐induced inflammatory activation and is sufficient to reduce the inflammatory and metabolic consequences of diet‐induced obesity (Koliwad *et al*., 2010). However, the effects of PPARγ activation on DGAT1 expression vary according to tissues with both increases and decreases in expression being reported in other cells (Shi *et al*., 2013).

Studies by Liu *et al*. (2012) showed that during monocyte differentiation, there is a metabolic switch towards glycolysis in the early initiation of inflammation in M1 cells and oxidative phosphorylation in M2 cells during the late adapting, resolving phases. More recently, a peptide mimicking ApoA1 that promotes cholesterol efflux from monocyte‐derived macrophages was shown to promote expression of genes required for oxidative phosphorylation with a concomitant increase in mitochondrial oxygen consumption (Datta *et al*., 2015). Our data show that polarization is differentially affected by the nature of FA. The SFA C16:0 polarized monocytes towards M1 macrophages, conversely, C18:1‐polarized monocytes to M2 macrophages that showed increased dependency on oxidative phosphorylation. Polarization towards M1 phenotype was associated with PPARγ inhibition, whereas polarization towards M2 associated with PPARγ activation. Incubating C16:0‐treated cells with rosiglitazone (PPARγ agonist) maintained PPARγ activity and decreased proinflammatory cytokine secretion. Conversely, knockdown of PPARγ in the C18:1‐treated monocytes elicited a more M1‐like polarization path with cells showing increased IL‐6 production and increasing dependency on glycolysis.

Together, these data suggest a mechanism whereby PPARγ is a controlling node for macrophage polarization and is sensitive to nutrients. A similar hypothesis is proposed in muscle, where control of acetylation serves as a critical node to control mitochondrial biogenesis, insulin sensitivity, glucose transport, ROS (Menzies & Auwerx, 2013). When NAD^+^ is depleted through increasing metabolism and uncoupling of the mitochondrial chain, the loss of SIRT activity will increase regulatory protein acetylation. Others have shown that PPAR acetylation affects its interaction with cofactors and other regulatory factors. For example, when deacetylated, its can no longer bind its inhibitor Dbc1 so promotes expression of brown adipose tissue genes, mitochondrial biogenesis genes and DGAT that favour lipid storage and oxidative phosphorylation (Qiang *et al*., 2012). When downregulated, its interaction with CBP is affected that in turn decreases NFkB activity and the secretion of TNF‐α is suppressed (Konstantinopoulos *et al*., 2007). However, when acetylated, for example when SFA are high and NAD^+^ is low, PPARγ activity is low, CBP interaction high and hence NFkB‐directed transcription is increased. NF‐kB/PPARγ expressional ‘on/off’ switches are common molecular events during colorectal carcinogenesis (Konstantinopoulos *et al*., 2007) and their wider importance in aging merits further investigation.

We have identified a FA profile that predicts inflammatory phenotype. Moreover, *de novo* ceramide synthesis and suppression of PPARγ drives monocytes towards an inflammatory macrophage phenotype in the presence of C16:0. This inflammatory phenotype predominantly relies on glycolysis for energy, an effect that can be mitigated by rosiglitazone and oleate (C18:1).

We suggest that in monocytes, the epigenetic control of PPARγ by altered nutrient profile controls the inflammatory phenotype. Our studies have identified a novel control node for inflammaging. This offers an explanatory mechanism of how diets rich in MUFA that lead to increased circulating MUFA such as the Mediterranean diet may be anti‐inflammatory via PPARγ activation. In addition, it offers the opportunity for developing selective modulators such as agonists that offer improved specificity to promote PPARγ activity and inhibit NFκB to support healthy aging (Xie *et al*., 2015).

## Experimental procedures

### Volunteers

Young male adults (18–35 years old; *n* = 26) and older male adults (50–70 years old, *n* = 21) were recruited for this study by local advertisement. Volunteers were healthy, nonsmokers and were not taking any disease modifying or anti‐inflammatory medication or nutritional supplements. Exclusion criteria were triglyceride < 2 mm and total cholesterol > 5.7 mm. Participants provided informed written consent, and ethical approval was obtained from the Aston University Ethics Committee.

### Blood measurements and processing

After an overnight fast, whole blood was drawn into EDTA between 8:00 and 10:30 hours. Anthropometric measurements were recorded. Biochemical indices (glucose, total cholesterol, HDL cholesterol and triglycerides) were measured using a Reflotron blood analyser (Boehringer Mannheim GmbH, Mannheim, Germany). Plasma insulin was determined by ELISA (Mercodia, Uppsala, Sweden). LDL cholesterol was calculated using the Friedewald equation. Plasma was prepared by centrifugation at 250 *g* for 10 min.

### FA analysis by gas chromatography

Analysis of the nonesterified FA profile profile of fasted plasma samples was adapted from Ichihara & Fukubayashi (2010) Briefly, after addition of internal standard C17 FA, lipids were extracted from 500 μL of plasma using chloroform methanol (2:1, 0.05% BHT) and subsequently centrifuged at 700 *g* for 10 min. The chloroform layer was dried under nitrogen and FA were methylated using 200 μL toluene, 1.5 mL methanol and 0.3 mL HCl in methanol at 100 °C for ~20 min in PTFE‐sealed glass vials. The FA methyl esters (FAMEs) were subsequently extracted with 1 mL of hexane and 1 mL of water, evaporated under nitrogen and resuspended in 50 μL of hexane prior to separation by GC using a HP‐Innowax polyethylene glycol polar column, with flame‐ionization detection. Identification of FA peaks was determined against peak areas of a Supelco 37 FAME mix standard.

### Conjugation of FA to BSA

Stock solutions of FA (200 mm) were prepared by dissolving their sodium salts into 0.1 m NaOH in 70% ethanol at 60 °C and conjugated to BSA as previously described (Gao *et al*., 2012).

### Cell culture

THP‐1 monocytes were maintained in RPMI 1640 with 10% foetal bovine serum at 37 °C in humidified 5% CO_2_/95% air incubator. To investigate the effect of FA on priming, THP‐1 monocytes (10^6^ mL^−1^) were pretreated with either FA (300 μm) or BSA control for 6 h prior to submaximal activation by LPS (5 ng mL^−1^) for a further 18 h. As previously described, cell viability was unaffected at these concentrations (Gao *et al*., 2012). As a positive control, maximal activation of THP‐1 cells was achieved using 200 ng mL^−1^ LPS. To investigate the effect of FA on polarity during differentiation into macrophages, THP‐1 monocytes (10^6^ mL^−1^) were pretreated with either FA (300 μm) or BSA control for 6 h prior to differentiation with PMA (320 nm) for a further 18 h. For inhibition experiments, THP‐1 monocytes were incubated with FB1 (50 μm), C18:1 (300 μm), 25 μm sirtinol and rosiglitazone (25 μm) in the presence or absence of C18:0 (300 μm). As a positive control for monocyte differentiation into M1 or M2 macrophages, THP1 monocytes were co‐incubated with cytokine cocktails for 24 h (100 ng mL^−1^ LPS and 20 ng mL^−1^ IFNγ for M1 or 20 ng mL^−1^ IL‐4 and 20 ng mL^−1^ IL‐13 for M2).

### Mitochondrial superoxide generation

Mitochondrial superoxide was determined in mononuclear cells immediately after blood collection. Whole blood that had been incubated with MitoSOX Red (1.25 μm; Invitrogen, Loughborough, UK) for 30 min exactly was immediately treated with Optilyse C prior to analysis by flow cytometry. For *in vitro* experiments, mitochondrial superoxide production was determined in differentiated THP‐1 macrophages after 24 h treatment using MitoSOX Red (1.25 μm; Invitrogen) after staining for 30 min.

### Glutathione determination

Total glutathione was measured using the dithionitrobisbenzoic acid recycling assay as described previously (Gao *et al*., 2012).

### Analysis of IL‐6, IL‐10, TNF‐α and TGF‐β1 cytokine levels

Circulating cytokines were determined by ELISA using undiluted plasma. To determine secreted cytokines during *in vitro* experiments, cells were treated with FA or control for 6 h and differentiated by PMA for 18 h. Cells were gently removed by pipetting and scraping. Cells were pelleted (100 *g*, 5 min) and cell‐free media containing cytokines were stored at −20 °C prior to analysis for IL‐6, IL‐10, TNF‐α (Peprotech, London, UK) and TGF‐β1 (R&D Systems, Abingdon, UK) by ELISA.

### Oil Red O staining for neutral lipids

Post‐FA or control treatments, 2 × 10^6^ THP1 cells were removed from multi‐well plates by gentle pipetting and scraping and were washed in phosphate‐buffered saline, then fixed with 4% w/v paraformaldehyde, and stained for 15 min with Oil Red O. Cells were then washed once in PBS, before extraction of stained lipids with isopropanol. Samples were centrifuged at 100 *g* for 2 min, with supernatants then transferred to a 96‐well plate and absorbance measured at 490 nm.

### Assessment of PPARγ activity

The activity of PPARγ in the nuclear fraction of THP1 cells was assessed using a TransAM PPARγ Transcription Factor Assay Kit (Active Motif, La Hulpe, Belgium) ELISA.

### Analysis of metabolic phenotype

Differentiated macrophages in 24‐well plates were centrifuged gently (40 *g*, 30 s) before removal of supplemented RPMI 1640. Medium was replaced by bicarbonate‐free PBS supplemented with 5 mm glucose, 2 mm l‐glutamine and 1 mm sodium pyruvate 1 h prior to analysis. Extracellular acidification rates and OCRs were measured under basal conditions and the MitoStress test using XF‐24 Extracellular Flux Analyzer (Seahorse Bioscience, Copenhagen, Denmark).

### SIRT1 activity analysis

THP‐1 cells were washed with ice‐cold PBS, lysed and the nuclear fraction collected by centrifugation according to the manufacturer's instructions. SIRT1 activity was measured in nuclear extract (15 μg of protein) using a kit purchased from Sigma‐Aldrich (Dorset, England, UK).

### Assessment NAD^+^:NADH ratio

The NAD^+^:NADH ratio in THP‐1 cells was assessed using a kit purchased from Abcam (Cambridge, UK).

### PPARγ RNA interference

Transient transfection with PPARγ‐siRNA was performed using lipofectamine RNA‐imax reagent according to a modified protocol and manufacturer's instructions. THP1 cells at approximately 50–70% confluence were transfected with siPPARγ or scrambled PPARγ‐siRNA (scr/siRNA) (Invitrogen). Cell culture medium was replaced at 6 h after transfection, and cells were then further incubated for 16–24 h before exposure to FA.

### SDS‐PAGE and western blotting

To confirm knockdown of PPARγ by siRNA, cells were lysed into RIPA buffer and 20 μg of each lysate in Laemmli buffer was separated by 10% SDS‐PAGE, transferred onto a PVDF membrane and blocked with 3% BSA. The membrane was probed with primary polyclonal anti‐PPARγ (1:1000; Santa Cruz, Dallas, USA) or monoclonal anti‐β‐actin (1:5000) for 2 h at room temperature followed by extensive washing then incubation with horseradish peroxidase‐labelled mouse anti‐rabbit IgG (1:15 000) or goat anti‐mouse IgG (1:20 000) for 2 h. The immunoreactive bands were detected by enhanced chemiluminescence reagent (GE HealthCare, Amersham, UK).

### Statistical analysis

Data analysed using spss version 21 (Armonk, New York, USA). Descriptive analysis of biochemical data from volunteers was performed for all variables with percentages, mean (standard deviation) and median (interquartile range), as appropriate. Baseline comparison between young and midlife group performed using appropriate statistical tests: chi‐squared tests for categorical variables, Student's *t*‐test for normally distributed continuous variables and Mann–Whitney *U*‐test for non‐normally distributed continuous variables. Univariate analysis was performed to determine the FA variables which were correlated significantly with age and markers of inflammation. These variables were then fitted into a multiple linear regression model by a backward stepwise method. Variables with a variance inflation factor > 10 and *F* statistic > 0.10 were removed from the next step of the model. Missing values were analysed pairwise.

For *in vitro* studies, statistical significance was determined by ANOVA with Tukey's post‐test using graphpad prism version 6 (La Jolla, California, USA).

## Funding

Biotechnology and Biological Sciences Research Council (Grant/Award Number: Ref: BB/G017832/1).

## Conflict of interest

None declared.

## Author contributions

CP and SS undertook experiments; CP JI, AB and ITN undertook data analysis; CP JEB, AD and CJB undertook interpretation and manuscript preparation; SJB recruited volunteers and analysed baseline characteristics; HRG undertook design, analysis and interpretation and authored the manuscript.

## Supporting information


**Table S1** Multiple linear regression model predicting plasma TNFα.
**Table S2** Multiple linear regression model predicting plasma IL‐6.
**Table S3** Multiple linear regression model predicting plasma TGFβ1.
**Fig. S1** Fatty acids do not elicit cytokine production from THP1 monocytes.
**Fig. S2** The inflammatory effects of saturated fatty acids are mitigated by NFkB inhibition and ROS scavenging.
**Fig. S3** Estimated daily dietary intake of fat in volunteers.

**Fig. S4** Transfection with siPPARγ knocks down PPARγ expression compared to scrambled siPPARγ.
