# Supplementary material for: Age‐associated changes in long‐chain fatty acid profile during healthy aging promote pro‐inflammatory monocyte polarization via PPARγ
Source: Aging Cell. 2015 Nov 2;15(1):128–39. doi: 10.1111/acel.12416 (PMC4717269; doi:10.1111/acel.12416)
Supplement: Supplementary file 1 — Table S1 Multiple linear regression model predicting plasma TNFα. Table S2 Multiple linear regression model predicting plasma IL‐6. Table S3 Multiple linear regression model predicting plasma TGFβ1. Fig. S1 Fatty acids do not elicit cytokine production from THP1 monocytes. Fig. S2 The inflammatory effects of saturated fatty acids are mitigated by NFkB inhibition and ROS scavenging. Fig. S3 Estimated daily dietary intake of fat in volunteers. [file ACEL-15-128-s001.docx]

**Supporting information**

**Table 1: Multiple linear regression model predicting plasma TNFα.** Fatty acid predictors of TNFα were adjusted for age, weight circumference and IL-6. Model summary: R= 0.810, R^2^= 0.656, Adjusted R^2^=0.609, P < 0.001

| Variable | Standardised coefficient  (β) | 95% CI | P |
| --- | --- | --- | --- |
| Intercept |  |  | 0.005 |
| C16:0 | 0.821 |  | 0.009 |
| IL-6 | 0.706 |  | <0.001 |
| C18:3n6 | -0.595 |  | 0.055 |

**Table 2. Multiple linear regression model predicting plasma IL-6.** Adjusted for age, waist circumference and IL-6. Model summary: R= 0.806, R^2^=0.649, Adjusted R^2^=0.562, P < 0.001

| **Variable** | **Standardised coefficient**  **(β)** | **P** |
| --- | --- | --- |
| Intercept |  | 0.489 |
| C18:3n6 | 0.598 | 0.129 |
| C24:0 | 0.291 | 0.12 |
| C14:0 | -0.253 | 0.114 |
| C18:3n3 | -0.641 | 0.08 |

**Table 3: Multiple linear regression model predicting plasma TGFβ1.** Adjusted for age, waist circumference and IL-10. Model summary: R = 0.699, R^2^= 0.489, Adjusted R^2^= 0.361, P < 0.05

| **Variable** | **Standardised Coefficient**  **(β)** | **P** |
| --- | --- | --- |
| **Intercept** |  | **0.004** |
| C18:0 | -0.552 | **0.006** |
| C24:0 | -0.613 | **0.019** |
| C18:3n6 | 0.657 | **0.003** |
| LDL cholesterol | -0.377 | **0.058** |
| Age | 0.453 | 0.069 |

**Supporting Figure 1. Fatty acids do not elicit cytokine production from THP1 monocytes**

**A B**


**C D**

**E F**

**Supporting figure 2.** The inflammatory effects of saturated fatty acids are mitigated by NFkB inhibition and ROS scavenging.

**Supporting Figure 3. Estimated daily dietary intake of fat in volunteers**.

Assessment of fat intake of mid-life volunteers (>50yrs; n=14) and younger volunteers (<30 years of age) using a three day estimated food diary, by DietPlan6 software. No significant difference in total fat intake was observed between the two groups.
