# Supplementary material for: Age‐associated changes in long‐chain fatty acid profile during healthy aging promote pro‐inflammatory monocyte polarization via PPARγ
Source: Aging Cell. 2015 Nov 2;15(1):128–39. doi: 10.1111/acel.12416 (PMC4717269; doi:10.1111/acel.12416)
Supplement: Supplementary file 2 — Fig. S4 Transfection with siPPARγ knocks down PPARγ expression compared to scrambled siPPARγ. [file ACEL-15-128-s002.pptx]

## Slide 1
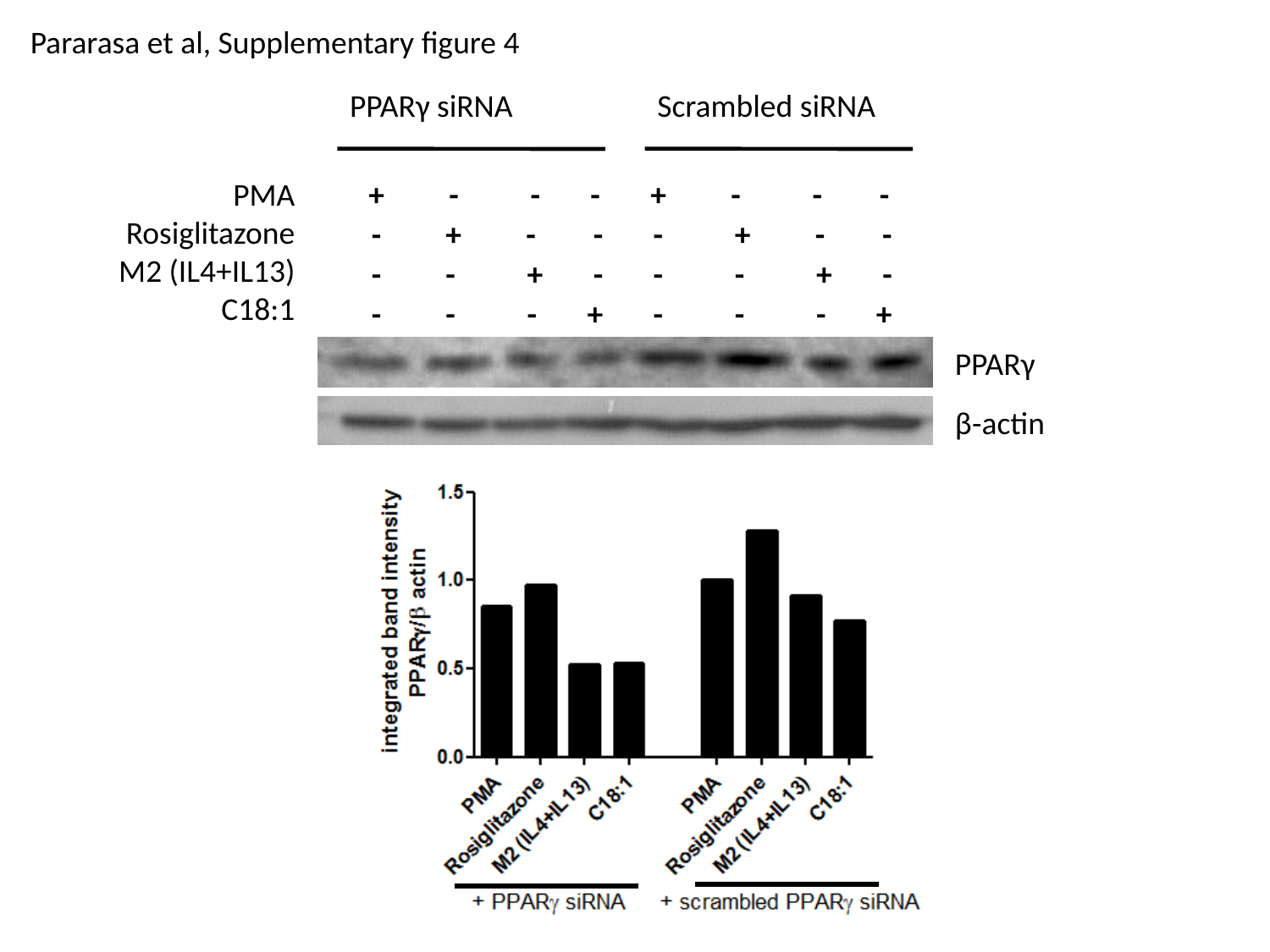

Pararasa et al, Supplementary figure 4
PPARγ siRNA
Scrambled siRNA
PMA
Rosiglitazone
M2 (IL4+IL13)
C18:1
 + - - - + - - -
 - + - - - + - -
 - - + - - - + -
 - - - + - - - +
PPARγ
β-actin
